# Stability of two competing populations in chemostat where one of the population changes its average mass of division in response to changes of its population

**DOI:** 10.1371/journal.pone.0213518

**Published:** 2019-03-27

**Authors:** Dimitrios Voulgarelis, Ajoy Velayudhan, Frank Smith

**Affiliations:** 1 Centre for Mathematics, Physics and Engineering in the Life Sciences and Experimental Biology, University College London, UCL, Physics Building, Gower Pl, London WC1E 6B, United Kingdom; 2 Department of Biochemical Engineering, University College London, Bernard Katz Building, Gordon Street, London WC1H 0AH, United Kingdom; 3 Department of Mathematics, University College London, Gower Street, London, WC1E 6BT, United Kingdom; Huazhong University of Science and Technology, CHINA

## Abstract

This paper considers a novel dynamical behaviour of two microbial populations, competing in a chemostat over a single substrate, that is only possible through the use of population balance equations (PBEs). PBEs are partial integrodifferential equations that represent a distribution of cells according to some internal state, mass in our case. Using these equations, realistic parameter values and the assumption that one population can deploy an emergency mechanism, where it can change the mean mass of division and hence divide faster, we arrive at two different steady states, one oscillatory and one non-oscillatory both of which seem to be stable. A steady state of either form is normally either unstable or only attainable through external control (cycling the dilution rate). In our case no external control is used. Finally, in the oscillatory case we attempt to explain how oscillations appear in the biomass without any explicit dependence on the division rate (the function that oscillates) through the approximation of fractional moments as a combination of integer moments. That allows an implicit dependence of the biomass on the number of cells which in turn is directly dependent on the division rate function.

## Introduction

### Motivation

Mathematical models of cell populations competing and coexisting in a bioreactor are almost as old as the experiments themselves. Bioreactors offer a unique opportunity to study phenomena associated with cell growth and their dynamics are so rich that have been studied for decades with still a lot of questions remaining unanswered. One of the most popular bioreactors for such explorations is the chemostat which is an automated bioreactor where spent medium which contains metabolic products, microorganisms and left over nutrients is continuously removed while fresh medium is added at the same rate to keep the volume constant [1]. That rate is called the dilution rate which when higher than the growth rate leads to extinction and otherwise to growth.

One of the most important aspects of competing cells with vast practical implication (waste-water treatment, ecology) is the coexistence of multiple populations. That particular aspect is one of the most widely studied and there have been many attempts to understand it. The main theorem here is the competitive exclusion principle (CEP) according to which when two or more populations are competing for the same source only one will survive. Which one depends on the break-even concentration defined as the concentration of the substrate that makes the growth rate equal to the dilution rate. Hence, the population with the smallest break-even concentration will win the competition. It has been shown that in the deterministic case populations that have the same break-even concentration can coexist [2] but that coexistence is unstable since when noise is included it collapses to a steady-state where only one population survives and the rest become extinct [3]. The only way that coexistence seems possible in deterministic systems is through the inclusion of delay [4] in the nutrient cycle or external control of the chemostat by periodically varying the dilution rate [5]. While mathematical models demonstrate the CEP in system of different population competing for a single source of food, nature has shown coexistence in these systems is possible which makes this aspect even more interesting and perplexing.

One of the reasons why the commonly used mathematical models in the chemostat might not be able to capture coexistence could be their simplicity. More complex mathematical models could perhaps be the key to new behaviours with as few as possible assumptions and external interventions. Population balance equations which have been used in engineering for decades offer the ability of modelling heterogeneity and its effects readily, by explicit modelling the internal state of the cells, whether that is age, mass, protein, DNA concentration or any intrinsic cell quality. Differences in these internal states mean differences in the behaviour of the cell and hence potentially richer dynamics.

### Aims

The aim of this paper is to investigate coexistence of two populations competing in a chemostat using PBEs and introduce the assumption of an “emergency mechanism”. Organisms can become more competitive in case of an imminent extinction through changes in the behaviour. Although this sort of adaptive behaviour is common in complex, multicellular organisms it seems to be the case for single cell ones. So in our model we have one population whose behaviour remains fixed and it is the one that under normal conditions out-competes and a second population that has a mechanism that allows it to change the mean division mass after sensing its population’s biomass. This sensing could be possible through a biomass dependent concentration of signal molecules that are secreted by the organism and are used for communication purposes. This is biologically relevant to a process called quorum sensing where bacterial cells communicate through signal molecules they release. Since the concentration of these molecules depends on the number of bacteria, it provides a representation of the total population or biomass and hence allows the cells to make population-density dependent decisions. Increase or decrease of these signalling molecules above or below a certain threshold leads to gene activation or depression and as a result physiological changes to the individual cells [6]. So in **Material and methods** we present the equations for the model as well as two variations to model the emergency mechanisms effect. One is mainly computational with a discrete change in the division rate and the other is equation-based and continuous. These will be used with and without delay in the sensing of the second organism. Additionally a stochastic version of the PBE is formulated that will be used for a steady-state stability investigation. In the **Results** the equations are solved numerically and simulated for both delay and non-delay cases and a parameter sweep is made to observe different steady-states. Moreover, numerical simulations of the steady-state and stochastic equations are performed to shed light on the coexistence steady-state. Finally, we attempt to explain the presence of oscillations in the biomass equations which are not explicitly dependent on the oscillating part (the division rate). Following the results section, our work and findings are summarized in the **Discussion** and in the last section named **Conclusion** we raise possible issues as well as possible extensions to our work.

## Materials and methods

### The model and parameters

Here we consider the case of two populations competing in a chemostat using a distribution model (PBE) and a non-trivial growth rate. Non-trivial growth rate refers to the use of a growth rate for the individual cells that depends on mass nonlinearly and does not allow for an easy retrieval of an ODE for the total biomass through the integration of the PBE model. The model equations are:

$$\begin{array}{cc} & \frac{\partial x}{\partial t}+\frac{\partial }{\partial m}\left(f\left(m,z\right)x\right)=2\int_{m}^{\infty }\Gamma_{x}\left(m^{\prime },z\right)p\left(m|m^{\prime }\right)xdm^{\prime }-\Gamma_{x}\left(m,z\right)x-Dx, \end{array}$$ (1)

$$\begin{array}{cc} & \frac{\partial y}{\partial t}+\frac{\partial }{\partial m}\left(g\left(m,z\right)y\right)=2\int_{m}^{\infty }\Gamma_{y}\left(m^{\prime },z\right)p\left(m|m^{\prime }\right)ydm^{\prime }-\Gamma_{y}\left(m,z\right)y-Dy, \end{array}$$ (2)

$$\begin{array}{cc} & \frac{dz}{dt}=D\left(z_{f}-z\right)-\frac{1}{Y_{1}}\int_{0}^{\infty }f\left(m^{\prime },z\right)xdm^{\prime }-\frac{1}{Y_{2}}\int_{0}^{\infty }g\left(m^{\prime },z\right)ydm^{\prime }. \end{array}$$ (3)

Here, we use the following notation; *x*, *y* are the cell distribution functions for the two cell populations, *z* is the substrate (oxygen) concentration, Γ is the division rate with the subscript defining which population it belongs to, *p* is the partition function for the dividing cell given by an asymmetric beta function to capture the asymmetrical division of cells. In practice the latter is almost always taken to be the same for both populations especially when dealing with similar cells (microbial in our case) as differences in division of different cells are usually reflected by differences in the division probability and rate. Finally *f*, *g* are the growth rate functions for the individual cells. The parameters of the model are defined in the Tables 1 and 2 below. The integrals are from 0 to infinity but cell masses above some maximum value (in our case 1) are extremely rare and the probability of not dividing by the time they reach them is almost zero. That plus the fact that an upper boundary is needed for numerical simulations is why for the remainder of the paper the upper limit will be given the value of 1.

**Table 1:** Parameters and their order of magnitude.

Parameter	Definition	Value
*D*	dilution rate (*h*^−1^)	varying *O*(10^−1^)
*α*	maximum rate of cell growth ng(cell mass)/*mm*^2^*h*^−1^	1.6 * 10^3^
*β*	half saturation constant mg(*O*_2_)/*L*	0.19
*ρ*	density of cells ng(cell mass)/*mm*^3^	1.14 * 10^6^
*z*_*f*_	substrate feed mg(*O*_2_)/*L*	0.15
*Y*	yield ng(cell mass)/mg(*O*_2_)	10^6^

**Table 2:** Numerical simulation parameters.

Parameter	Definition	Value
*D*	dilution rate (*h*^−1^)	0.31
*α*_1_	maximum rate of cell growth ng(cell mass)/*mm*^2^*h*^−1^ of population x	2000
*α*_2_	maximum rate of cell growth ng(cell mass)/*mm*^2^*h*^−1^ of population y	1280
*β*_1_	half saturation constant mg(*O*_2_)/*L* of population x	0.2
*β*_2_	half saturation constant mg(*O*_2_)/*L* of population y	0.12
*ρ*	density of cells ng(cell mass)/*mm*^3^	1.14 * 10^6^
*z*_*f*_	substrate feed mg(*O*_2_)/*L*	0.18
*Y*_1,2_	yield ng(cell mass)/mg(*O*_2_)	10^6^

The growth rate functions *f*, *g* are variations of the classic spherical cell growth model since we assume that no mass is lost due to catalytic reactions. So both the growth rates and the general form of the PBE used is similar to that proposed by Eakman et. al (1966) [7].

$$\begin{array}{cc} f(m,z) & ={\left(\frac{36\pi }{\rho^{2}}\right)}^{1/3}\frac{\alpha_{1}zm^{2/3}}{\beta_{1}+z}, \end{array}$$ (4)

$$\begin{array}{cc} g(m,z) & ={\left(\frac{36\pi }{\rho^{2}}\right)}^{1/3}\frac{\alpha_{2}zm^{2/3}}{\beta_{2}+z}. \end{array}$$ (5)

This form of the growth rate is both realistic and allows for different dynamics from the case where the PBE can be integrated back to the ODE for the biomass. That is what from now on we will call the trivial case in which the growth rate has the form:

$$\begin{array}{cc} f_{T}\left(m,z\right) & =\frac{\alpha_{1}zm}{\beta_{1}+z}, \end{array}$$ (6)

$$\begin{array}{cc} g_{T}\left(m,z\right) & =\frac{\alpha_{2}zm}{\beta_{2}+z}. \end{array}$$ (7)

In that case it can be shown that if we multiply Eqs (1) and (2) by m and integrate through the whole mass spectrum, the birth and death terms cancel out and we are left with an ODE for the biomass $N_{b}^{x}$ defined as:

$$\begin{array}{c} N_{b}^{x}=\int_{0}^{1}xmdm. \end{array}$$ (8)

The biomass ODE is the familiar Chemostat ODE:

$$\begin{array}{cc} \frac{dN_{b}^{x}}{dt} & =N_{b}^{x}\left(\frac{\alpha_{1}z}{\beta_{1}+z}-D\right), \end{array}$$ (9)

$$\begin{array}{cc} \frac{dN_{b}^{y}}{dt} & =N_{b}^{y}\left(\frac{\alpha_{2}z}{\beta_{2}+z}-D\right), \end{array}$$ (10)

$$\begin{array}{cc} \frac{dz}{dt} & =D\left(z_{f}-z\right)-\frac{1}{Y_{1}}N_{b}^{x}\frac{\alpha_{1}z}{\beta_{1}+z}-\frac{1}{Y_{2}}N_{b}^{y}\frac{\alpha_{2}z}{\beta_{2}+z}. \end{array}$$ (11)

In Table 1 we define the parameters for the PBE model and give some experimental values as found in [8, 9] for cells absorbing oxygen converted to appropriate units.

**Fig 1:**
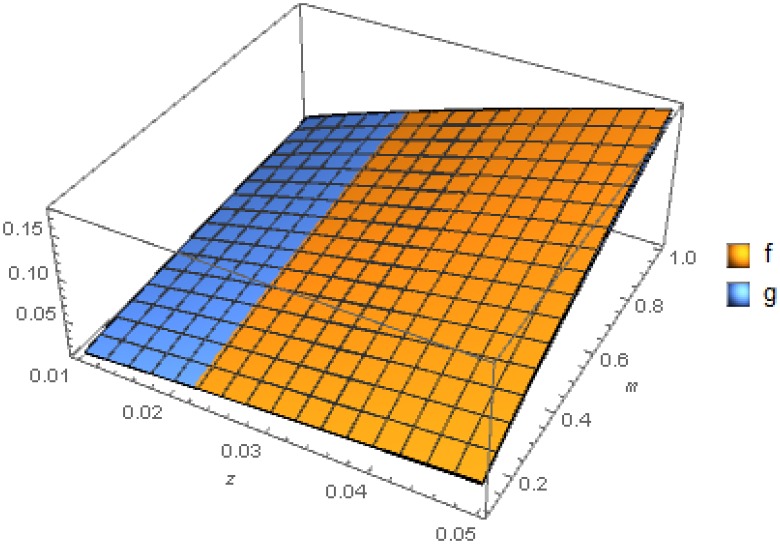
Growth rates *f* and *g* plotted against individual cell mass and substrate concentration. The parameter values selected were such that there is an intersection curve creating two regions where a different population has an advantage.

The next table, Table 2, summarises the parameters chosen for the numerical simulations of two population competing. The parameters are realistic variations of the values referenced above and are chosen such that *f*, *g* have the following intersection, see Fig 1. We wanted the an intersection to occur in order to have both population having an competitive advantage on different regions of the parameter space used rather than having one dominating throughout.

Fig 1 shows that for the mass and substrate space has been divided into two regions, one where cells of population x have higher growth rates and one where y cells do. This was done so that we do not have one type of cell dominating throughout which could lead to the trivial result of one population always out-competing the other.

In the case of modelling the competition with ODEs the intersections are distinct points whereas in this case, as can been seen in Fig 1, it is a curve. Moreover the deterministic result for competition using an ODE is well explored and understood. Our aim is to determine whether we can achieve different dynamics using the features only available in PBEs and not ODEs. Initially we can see that there is only a single case where the PBE can be integrated back to the classical ODE with Monod kinetics for the competition of populations in a chemostat and that is when *f*, *g* have the form of linear dependence on the mass defined in (6) and (7) and referred to as “trivial”.

If we multiply the PBE, using this form of growth rates, with *m* and then integrate with respect to it we retrieve the well known ODE models and the stability analysis performed in Stephanopoulos et al can be performed. Instead of the above form we will use Eqs (4) and (5) so that we cannot integrate the PBE back to the ODE.

### Changing the average mass of division as an emergency mechanism

It is easy to show that simulating the PBE with the above parameters is not very interesting on each own since only one population will survive and it will be the same one every time. We want to explore a special cases of limit cycle which can only occur when using a PBE. We postulate the cells of population x divide normally at a high average mass of 0.75*ng* whereas the cells of population y have the ability to change the average mass of division according to the changes of their total biomass. More specifically if the population drops below a critical value the y population cells will start changing their internal mechanism so they can divide at a lower average mass of 0.4*ng* but if their biomass increases above that value they will return to the same average mass of division as cells from population x. Furthermore we make the simplifying assumption that cells can very quickly sense if their population has crossed a critical value but from detection to response it takes 7 days. Hence there is a delay since cells would possibly require some time to change their internal mechanisms leading to a different average mass of division that before. That difference of mean mass of division comes from changing the value at the division probability found in the division rate Γ. The division rate is given by:

$$\begin{array}{c} \Gamma_{x}=\frac{h(m)}{1-\int_{0}^{m}h\left(m^{\prime }\right)dm^{\prime }}f\left(m,z\right), \end{array}$$ (12)

where, *h*(*m*) is a Gaussian representing the probability of division with mean, *m*_0_, as the mean mass of division.

$$\begin{array}{c} h\left(m\right)=\frac{1}{\sqrt{2\pi \sigma^{2}}}e^{-\frac{{\left(m-m_{0}\right)}^{2}}{2\sigma^{2}}}. \end{array}$$ (13)

The division probability is maximum at the mean mass of division and afterwards is decreasing but the rate is increasing and approaching infinity as the cells approach the upper mass limit. Hence, the rate of division for very large cells is very high, making it impossible for them not to divide before reaching the maximum value.

**Fig 2:**
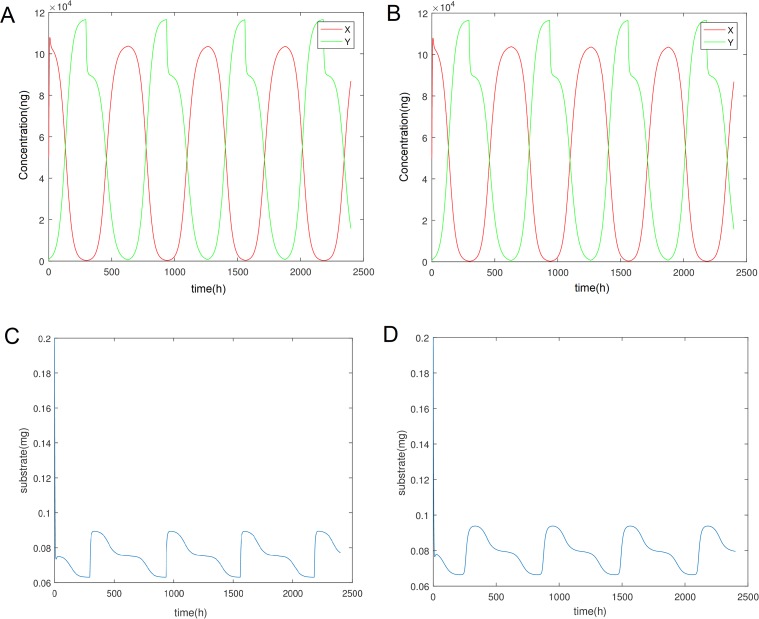
Limit cycle of both the competing populations and the substrate for Eqs (1)–(3). (A) Oscillations of the two biomasses for populations X,Y (Discrete). (B) Oscillations of the two biomasses for populations X,Y (Continuous). (C) Oscillation in the concentration of *O*_2_ (Discrete). (D) Oscillation in the concentration of *O*_2_ (Continuous).

Two models for this process were created that are exactly equivalent, see Fig 2. Both are equation based but in the first the switching happens discretely between two different Γ_*y*_ function for population y through an IF statement and the delay is also implemented computationally. The equations for both models are (1)–(5) with the first using (12) and (13) whereas the second has (13) and (14). For the second model we made the process continuous and the delay is built in the model. Equations for *x* and *z* remain the same but the equation for *y* is altered by changing Γ_*y*_ to the following:

$$\begin{array}{c} \Gamma_{y}\left(m,z\right)=[1+\frac{Y_{c}-Y_{t-168}}{\sqrt{K+{\left(Y_{c}-Y_{t-168}\right)}^{2}}}]\frac{\Gamma_{y}^{1}}{2}+[1+\frac{Y_{t-168}-Y_{c}}{\sqrt{K+{\left(Y_{t-168}-Y_{c}\right)}^{2}}}]\frac{\Gamma_{y}^{2}}{2}. \end{array}$$ (14)

Here, the superscript indicates the different division rate due to the different mean mass of division and the rest is just a double sigmoidal function that makes the first term dominate under the critical population denoted by *Y*_*c*_ and the second term dominate above. For the purposes of the delay we have assumed that the populations are kept at a constant concentration for the first seven days. *Y*_*t*−168_ represents the value of the biomass of population y seven days ago (168 hours) and is the delayed value sensed by the cells.

The two models were solved numerically using finite differences and the results were validated with the use of a different numerical method, namely orthogonal collocations.

Finally, for the equation based model with the composite Γ_*y*_, we explore the case without the delay as well as its steady-state. In order to validate the existence of the steady-state numerically as well as explore its stability we solve numerically the steady-state model, by setting the time derivative in (1)–(3) to zero, i.e. $\frac{\partial x}{\partial t}=\frac{\partial y}{\partial t}=\frac{dz}{zt}=0$, and a stochastic PBE with a general multiplicative noise. The stochastic equations are given by:

$$\begin{array}{cc} & dx=dt(-\frac{\partial }{\partial m}\left(fx\right)+2\int_{m}^{1}\Gamma_{x}\left(m^{\prime },z\right)p\left(m|m^{\prime }\right)xdm^{\prime }-\Gamma_{x}x-Dx)+\sigma_{1}xdW_{1}, \end{array}$$ (15)

$$\begin{array}{cc} & dy=dt(-\frac{\partial }{\partial m}\left(gy\right)+2\int_{m}^{1}\Gamma_{y}\left(m^{\prime },z\right)p\left(m|m^{\prime }\right)ydm^{\prime }-\Gamma_{y}y-Dy)+\sigma_{2}ydW_{2}, \end{array}$$ (16)

$$\begin{array}{cc} & dz=dt(D\left(z_{f}-z\right)-\frac{1}{Y_{1}}\int_{0}^{1}fxdm^{\prime }-\frac{1}{Y_{2}}\int_{0}^{1}gydm^{\prime })+\sigma_{3}zdW_{3}. \end{array}$$ (17)

where, *dW*_*i*_ are the Weiner increments of three independent Wiener processes *W*_1_, *W*_2_, *W*_3_ and *σ*_*i*_ are the noise intensities.

The steady equations were solved using finite differences and an iterative Newton-Raphson method whereas the stochastic equations were solved by discretizing the mass derivative and then using the Euler-Maruyama algorithm.

The boundary condition used in all simulation of the PBE are the containment conditions *f*(0, *z*)*x*(0, *t*) = *f*(1, *z*)*x*(1, *t*) = *g*(0, *z*)*y*(0, *t*) = *g*(1, *z*)*y*(1, *t*) = 0. The initial conditions for all PBE simulations are Gaussian distributions with mean *μ* = 0.5 and standard deviations *σ* = 0.0375 multiplied by 10^5^, 3 * 10^3^ for x and y respectively to give the initial biomass.

## Results

### Oscillatory steady state

Simulating the discrete model for Eqs (1)–(3) for 100 days and with the parameters stated before we obtain the following behaviour for the biomass of the two populations and the substrate:

What we observe in Fig 2 is that the biomass of the two cell populations for both formulated models exhibits an oscillatory behaviour which seems to remain regular throughout the simulation and throughout longer simulations not shown here. Hence we come to the conclusion that the system has reached a limit cycle steady state. In addition to the cell biomass, the concentration of the substrate is also admitting oscillations of the same frequency. It can be seen clearly that the two models (discrete and continuous) have almost identical dynamics with the exception of sharp transitions in the case of the discrete mechanisms change. Hence for the subsequent analysis we will use the discrete case as it is more computationally efficient.

**Fig 3:**
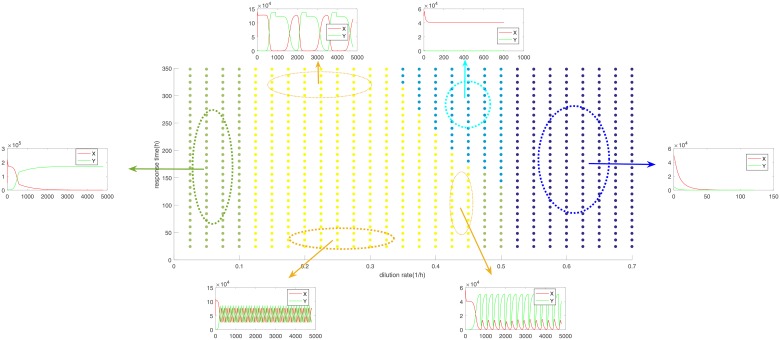
Parameter space behaviour of the discrete model for varying values of the dilution rate and response time. Different colours correspond to different steady states with extinction of population y and steady state for x (light blue), extinction of x and steady state for y (light green) and finally coexistence through oscillations (yellow).

To shed further light into the dynamics of our model we varied two key parameters, the time of response and the dilution rate to see what are the different dynamics obtained as well as determine whether the oscillatory steady-state is robust to changes in these two parameters. An algorithm was produced to classify the behaviour into 4 different categories. These are extinction of both populations (dark blue), extinction of population y and steady state for x (light blue), extinction of x and steady state for y (light green) and finally coexistence through oscillations (yellow). Fig 3 shows the parameter space along with plots of the dynamics in different areas of the parameter space.

From the plot above we can see that the limit cycle steady state occurs for a range of different values and seems to be stable as a similar plot is obtained for a different choice of growth parameters. This type of behaviour has been observed before in the context of a chemostat where externally induced oscillation of the substrate cause the concentrations of the competing populations to oscillate [4]. What is unique about our system is that the same phenomenon is now induced completely internally, from the system itself, which can only be captured with the use of PBEs. Moreover, even within the same steady-state region varying these parameters can lead to different behaviours. Focusing more on the interesting yellow area we see that the frequency of oscillation depends heavily on the response time where faster response means more oscillations. Increasing the dilution rate seems to provide two contrasting results depending on the response time. For faster response times increasing the dilution rate gives an advantage to y and can even lead to x extinction whereas the opposite is true for slower response times.

### Non-oscillatory case

Although delays provide a more realistic approach it is interesting to explore what happens when no delay is included. The Γ function remains the same as that defined previously but the sensing of the biomass happens without delay for population y. Simulation of this system leads to a non-oscillatory steady state where both populations survive at a fixed value.

**Fig 4:**
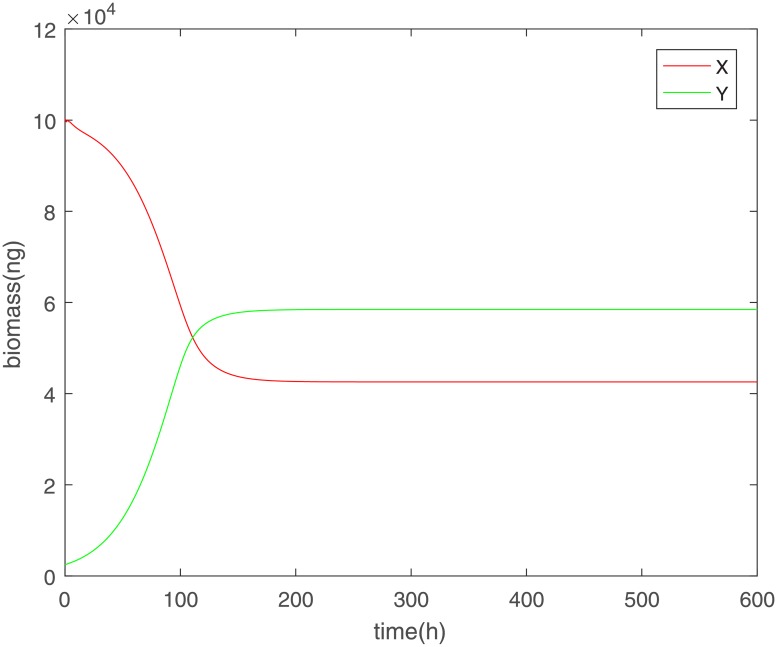
Evolution and steady state of competing populations without delay in the sensing of the biomass by population y but the same parameters as in the previous simulations.

**Fig 5:**
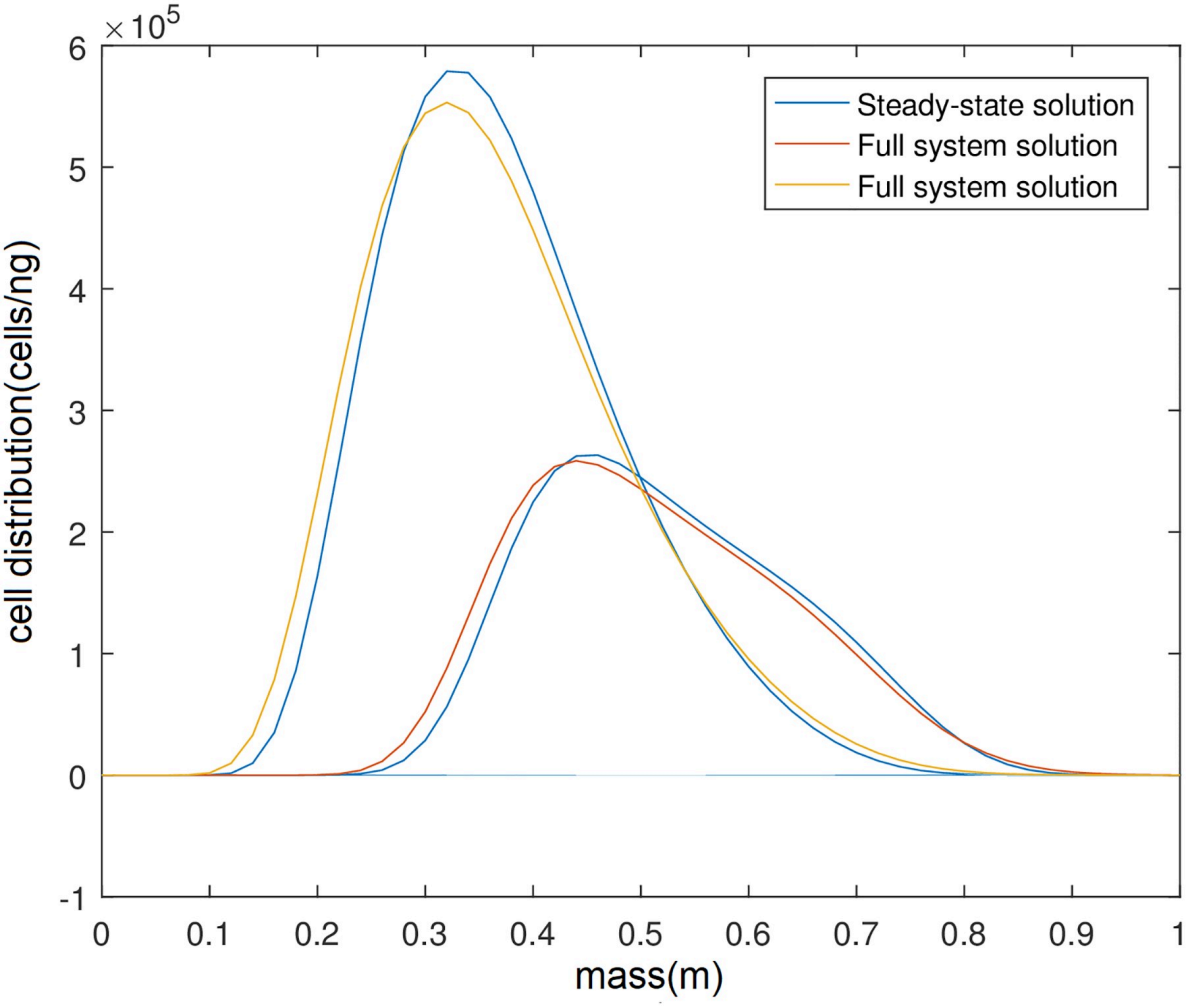
Steady state distribution of competing populations x and y from numerical solution of the steady-state form of Eqs (1)–(3) (zero time derivative) in yellow and red respectively and from the numerical solution of the full system of Eqs (1)–(3) in blue. For all simulations we assume that there is no delay between change detection and response.

In Fig 4 we can see that after an initial period both populations reach a non-oscillatory state with a steady biomass level. To confirm whether that is indeed a true steady-state and not a numerical artefact we compared the results of the full system numerical solutions to the steady-state system numerical solution seen in Fig 5.

Solving numerically the steady-state equations yields a result that matches the full system very closely even when the initial guess is far from the steady-state, see Fig 5. After many iterations of the steady-state version of (1)–(3) the distributions of the two populations shown in yellow and red reach a fixed value which fits closely the distribution arrived by the numerical solution of the full system (blue). That seems to be the case for many different initial guesses of the iteration process, even some that are far off the final solution.

As a result we end up with very similar biomass values for the two different solution which leads us to believe that we are dealing with a true steady-state. The next question is what is the stability of the steady-states and hence how will it behave under noise. It is well documented that deterministically there can be a steady-state with both populations at a fixed concentration in the chemostat [2] which turns out not to be stable under the influence of noise [10]. So we set out to find whether that is the case here. To that end we simulate the set of stochastic Eqs (16)–(18). Since analysis of the steady state analytically is not readily done, we hoped that the numerical simulations of the stochastic system would provide some indication.

**Fig 6:**
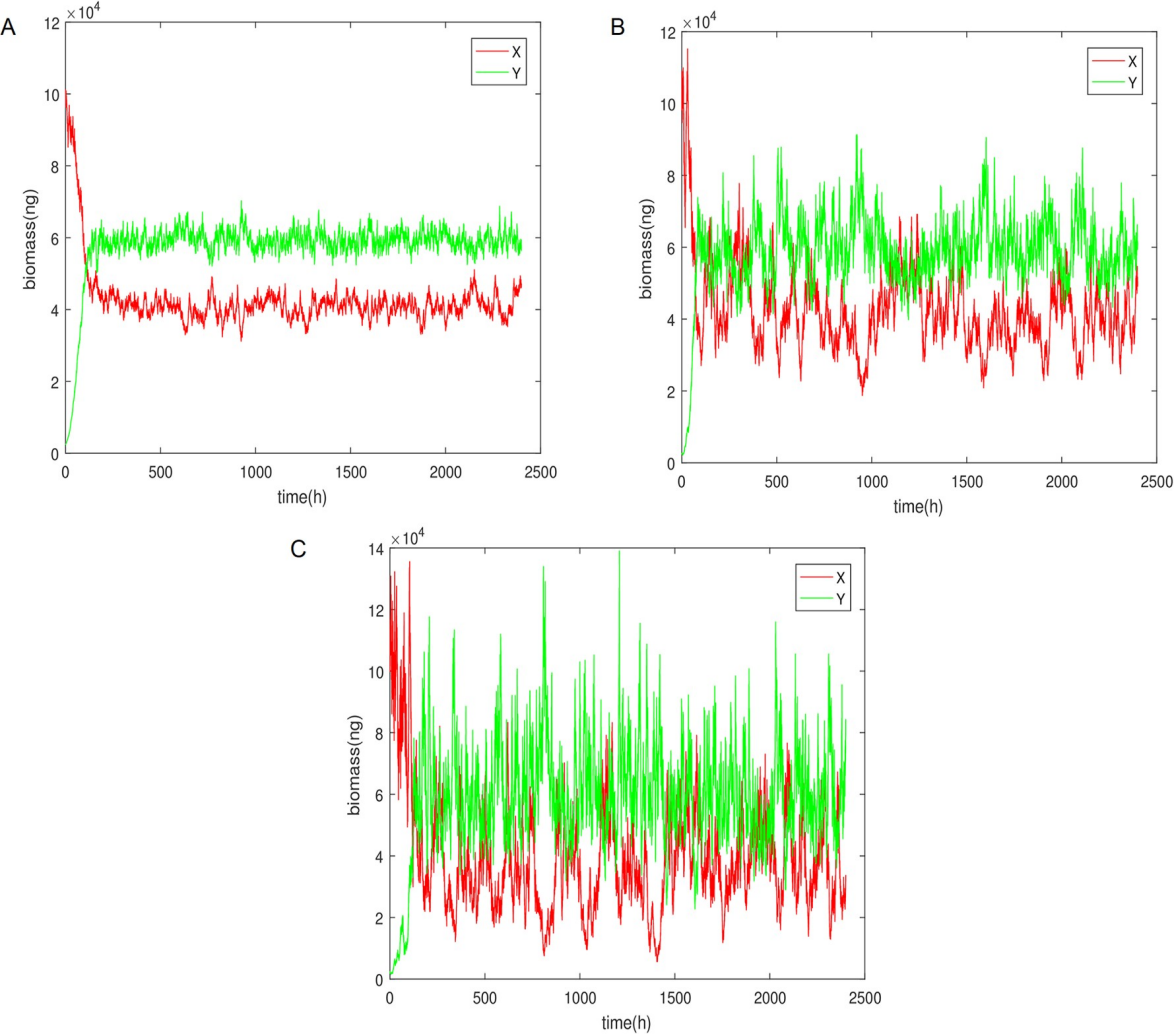
Stochastic simulation of the biomass of the the two populations, using the Euler-Maruyama method, for low, medium and high noise intensity values equal for both population in all simulations. (A) *σ*_1_ = *σ*_2_ = *σ*_3_ = 0.02. (B) *σ*_1_ = *σ*_2_ = *σ*_3_ = 0.05. (C) *σ*_1_ = *σ*_2_ = *σ*_3_ = 0.1.

As you can see in Fig 6 with the addition of noise the system varies around the steady-state but does not converge to a different one. Of course that result might be dependent on the amount of noise as well as the specific form of Γ_*y*_ but for reasonable noise intensity levels it tends to make the case that the steady state is indeed stable. The noise intensities were assumed to be the same for both populations and substrate because there is no apparent reason to believe it needs to be different.

### Source of oscillations in the biomass

It is very interesting to notice that when the oxygen oscillates, it does not cross the critical value where the individual growth rates of cells of population y are greater than the one of cells of population x. That raises the question as to what causes this oscillation and sustained competition between the two cell types. To this end let us first derive the equation for the biomass and then the total number of cells. For the first we need the first moment of Eq (1) and for the second the zeroth moment. First we define the biomass and total number of population x as:

$$\begin{array}{cc} N_{b}^{x}\left(t\right) & =\int_{0}^{1}mx\left(m,t\right)dm, \end{array}$$ (18)

$$\begin{array}{cc} N_{T}^{x}\left(t\right) & =\int_{0}^{1}x\left(m,t\right)dm, \end{array}$$ (19)

Then we find that the equations for these quantities are given by:

$$\begin{array}{cc} {\dot{N}}_{b}^{x}\left(t\right) & =\int_{0}^{1}f\left(m,z\right)xdm-DN_{b}^{x}, \end{array}$$ (20)

$$\begin{array}{cc} {\dot{N}}_{T}^{x}\left(t\right) & =\int_{0}^{1}(2\int_{m}^{1}\Gamma_{x}\left(m^{\prime },z\right)p\left(m|m^{\prime }\right)xdm^{\prime }-\Gamma_{x}\left(m,z\right)x)dm-DN_{t}^{x} \end{array}$$ (21)

$$\begin{array}{cc} & =\int_{0}^{1}\Gamma_{x}\left(m,z\right)xdm-DN_{t}^{x} \end{array}$$ (22)

where to derive the above we used the fact that the total divisions and births cancel out in the biomass equation and the containment condition, i.e. *f*(*m*, *z*)*x*(*m*, *t*) = 0 at the boundaries, for the total number of cells.

Using Eq (18) and inspired by the ODE Eq (8) we define the following functions which we will call the biomass growth rates:

$$\begin{array}{cc} F & =\frac{\int_{0}^{1}f\left(m,z\right)xdm}{N_{b}^{x}}, \end{array}$$ (23)

$$\begin{array}{cc} G & =\frac{\int_{0}^{1}g\left(m,z\right)ydm}{N_{b}^{y}}. \end{array}$$ (24)

In the trivial case these are given by the Monod function shown in Eqs (8) and (9) but in the non-trivial cases we cannot analytically derive them and as a result they might not be just a function of the substrate. What we can do is plot them numerically. In our numerical example the plot is as follows.

**Fig 7:**
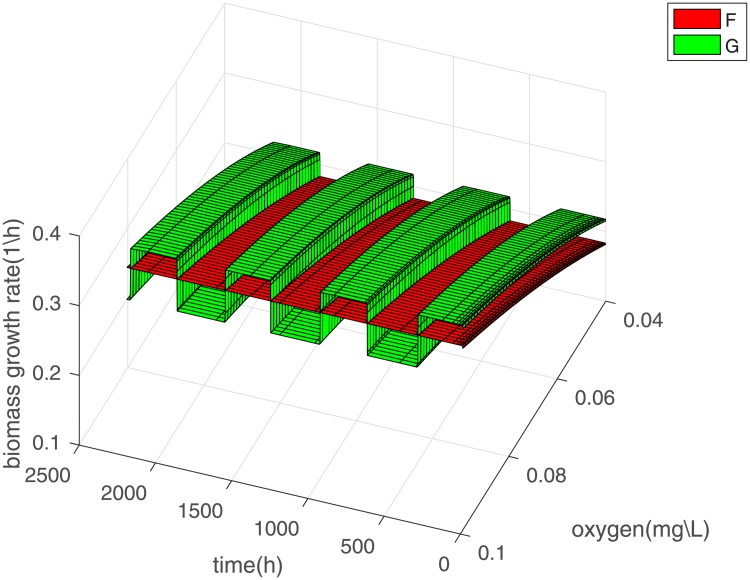
Biomass growth rate functions F, G, defined in (23) and (24), plotted for fixed time and varying values of the substrate and for fixed values of the substrate and varying time to observe the form of the functions which, as can be seen from the definition, are oxygen dependent.

**Fig 8:**
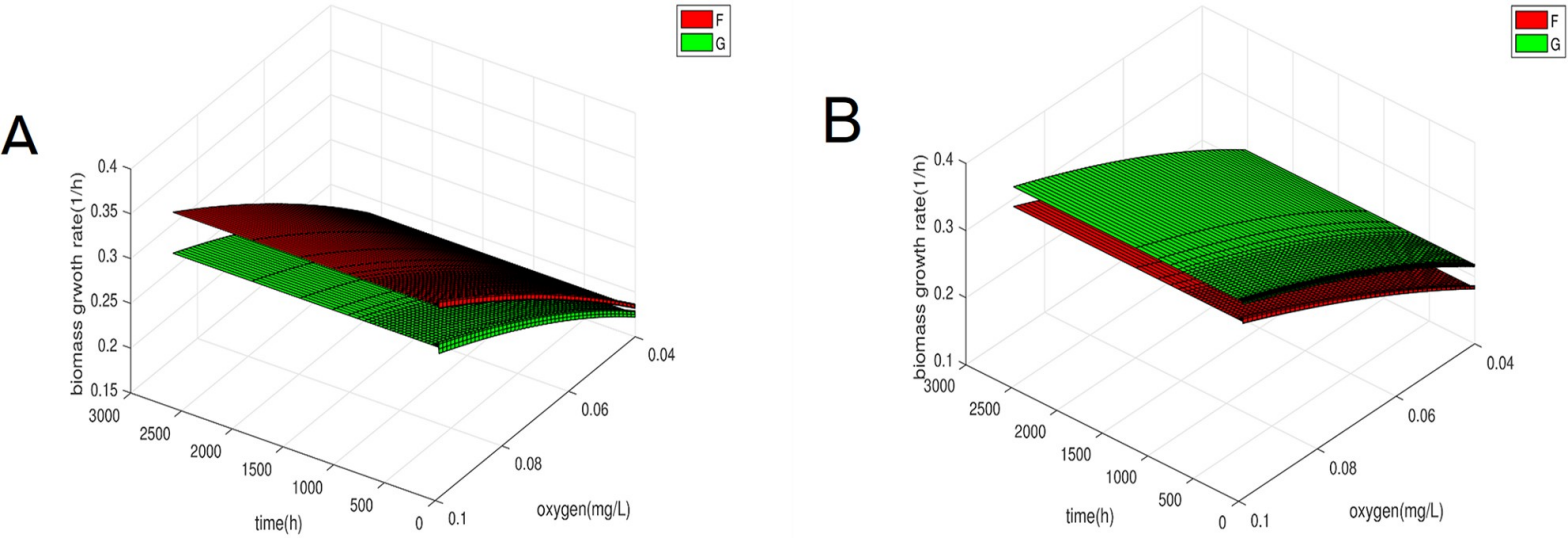
Biomass growth rate G (24) for different average mass of division for population y but fixed throughout the simulation. (A) Biomass growth rate for average mass of division of both populations at 0.75. (B) Biomass growth rate for average mass of division 0.4 for population y and 0.75 for population x.

In Fig 7 we calculated the functions F,G for different times during the model and then plotted them for different values of oxygen concentration. It is very interesting to observe that function the F has the same Monod-like form throughout the simulation whereas the function G changes its form as Γ_*y*_ is changed and oscillation are two Monod-like forms. Furthermore we can see that for the range of values where the oxygen concentration oscillates either F or G is higher which could justify the oscillations in the biomass. Moreover if we simulate the model again but keeping the average mass of division fixed once at a value of 0.75 and the other 0.4 for population y, i.e. the values between which it oscillates in the original case, we find that *G* has a different form in each case which is constant and corresponds to the upper and lower forms of the oscillatory case, as seen in Fig 8. In the first case y dies out and x survives whereas the opposite happens in the second case as expected by the initial results.

All the above means that there is some dependence of the biomass on the division rate Γ in the non-trivial case. That is counter-intuitive as there is no explicit dependence in the equations. So the question is where is that dependence coming from. What could help us understand this is simulating and plotting the “biomass growth rates” for different powers of the mass variable between zero and one. One is the trivial case were we can retrieve the ODE. So we will vary n in the functions below:

$$\begin{array}{cc} f_{n}\left(m,z\right) & ={\left(\frac{36\pi }{\rho^{2}}\right)}^{1/3}\frac{\alpha_{1}zm^{n}}{\beta_{1}+z}, \end{array}$$ (25)

$$\begin{array}{cc} g_{n}\left(m,z\right) & ={\left(\frac{36\pi }{\rho^{2}}\right)}^{1/3}\frac{\alpha_{2}zm^{n}}{\beta_{2}+z}. \end{array}$$ (26)

**Fig 9:**
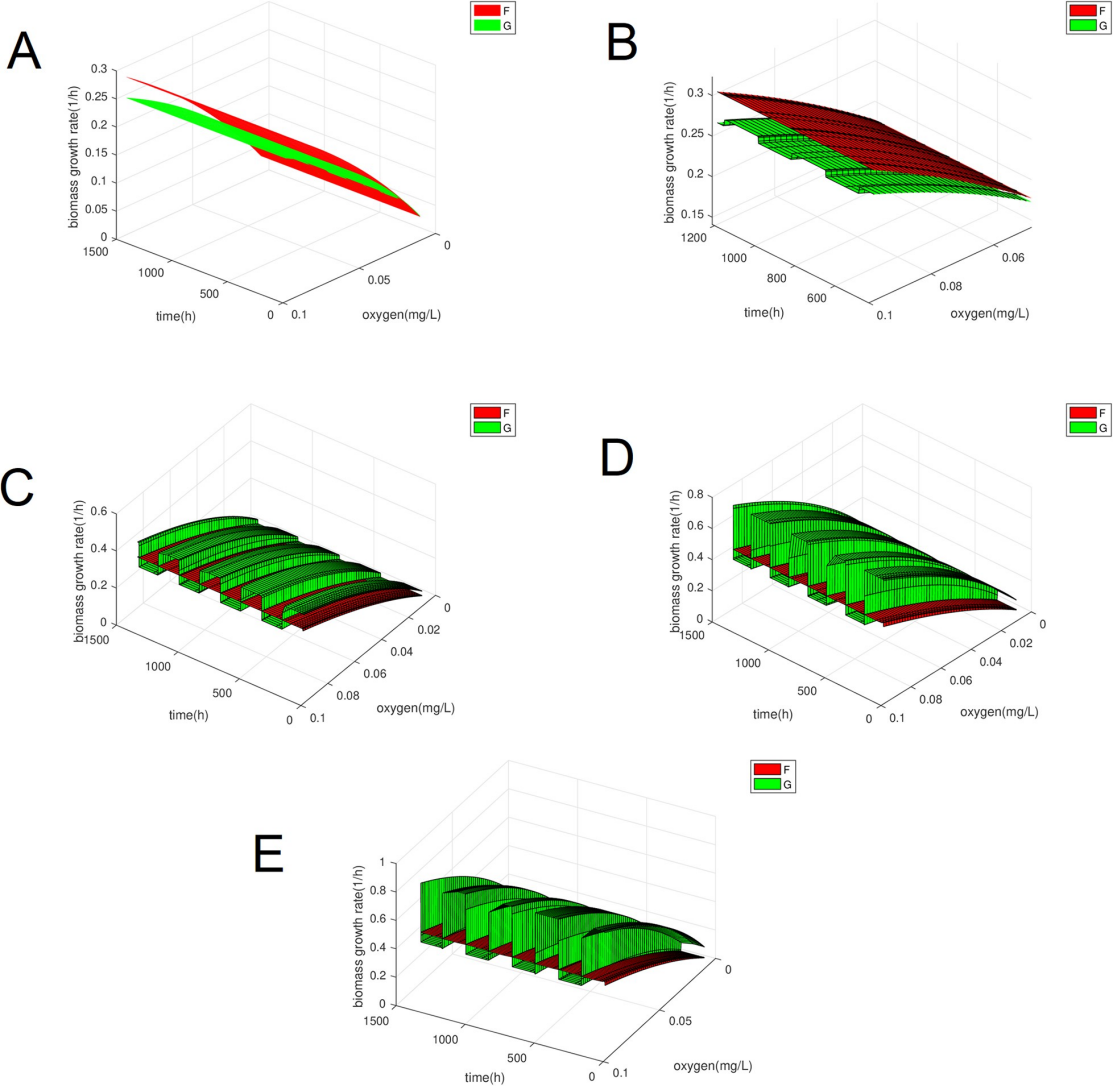
Biomass growth rates F,G for 5 different values of the power of the mass variable, n, in the individual growth rate functions. (A) Biomass growth rates for n = 1. (B) Biomass growth rates for n = 0.95. (C) Biomass growth rates for n = 0.5. (D) Biomass growth rates for n = 0.1. (E) Biomass growth rates for n = 0.

Obviously for many values of n these growth function will not make physical sense but we are just looking for a connection between the appearance of Γ dependence in the biomass Eq (20) as we depart from the trivial case. So we simulate and plot five cases, *n* = 1, *n* = 0.95, *n* = 0.5, *n* = 0.1, *n* = 0 in Fig 9. In the first case we expect to see no oscillations despite the changing of Γ_*y*_ since we can derive an analytical formula for the biomass. Moreover we expect that when *n* is close to one the oscillations are smaller and increase the further n is away from 1 until reaching a maximum range. To check whether or not there are oscillations in the biomass growth rates we will force the average mass of division of y and hence Γ_*y*_ to change automatically after 7 days independent of satisfying the biomass condition we have enforced in the initial simulation.

As expected we see no oscillation in the *n* = 1 case despite the oscillations in the average mass of division and as *n* decreases the oscillations increase. Although we cannot derive a non-integral ODE for the biomass in the cases where 0 < *n* < 1, we can in the case where *n* = 0. Let us take the equations for the x population. The individual growth rate has no dependence on m and so we can take it out of the integral in Eq (20) which just leaves the total number of cells.

$$\begin{array}{c} {\dot{N}}_{b}^{x}={\left(\frac{36\pi }{\rho^{2}}\right)}^{1/3}\frac{\alpha_{1}z}{\beta_{1}+z}N_{t}^{x}-DN_{b}^{x} \end{array}$$ (27)

So, the biomass is dependent on the total number of cells whose equation is given by (21). The total number of cells depends explicitly on the division rate and due to (26) so does the biomass. Despite the fact that this is true in the case of *n* = 0 it points us towards thinking that in the rest of the cases where *n* < 1 we obtain some dependence of the biomass on the total number of cells which explains the dependence on Γ and hence the oscillations.

A formal answer as to why this is the case comes in the form of fractional moments. Fractional moments are the non-integer moments and an approximation was found in [11] that can relate them to integer moments using the Weyl fractional derivative and the moments generating function. It is known that the kth derivative of the moments generation function gives the kth moment of a distribution. According to [11] a fractional moment can be approximated as a sum of the N integer moments by the formula:

μα=∑i=0n(αi)(∑j=0i(-1)i+j(ij)Uα-jμj), (28)

where, *U* is related to the mean and standard deviation of the distribution through *U* = *M* + *βσ*, given by:

$$\begin{array}{c} U=\frac{\mu_{1}}{\mu_{0}}+\beta \sqrt{\mu_{2}+\mu_{0}{\left(\frac{\mu_{1}}{\mu_{0}}\right)}^{2}}. \end{array}$$ (29)

The choice of *β* determines what part of the distribution we will use and represents the removal of the tail of the distribution [11] as with the above approximation it is assumed:

$$\begin{array}{c} \mu_{k}=\int_{0}^{\infty }m^{k}f\left(m\right)dm\approx \int_{0}^{U}m^{k}f\left(m\right)dm. \end{array}$$ (30)

In our case we can see that from Eq (18) we have the following fractional moment:

$$\begin{array}{c} \mu_{2/3}^{x}=\int_{0}^{1}m^{2/3}x\left(m,t\right)dm \end{array}$$ (31)

Similarly for y.

It follows from (25) that no matter how many moments we choose to include in our approximation there will always be a dependence on the zeroth moment and hence the number of cells which as stated is explicitly dependent on the division rate. To confirm that this approximation is indeed valid we have plotted the fractional moment $\mu_{2/3}^{y}$, both directly from the distribution function as well as Eq (28) using only the zeroth, first moment and second moments. We have named them respectively $\mu_{2/3}^{y}$ and ${\hat{\mu }}_{2/3}^{y}$.

**Fig 10:**
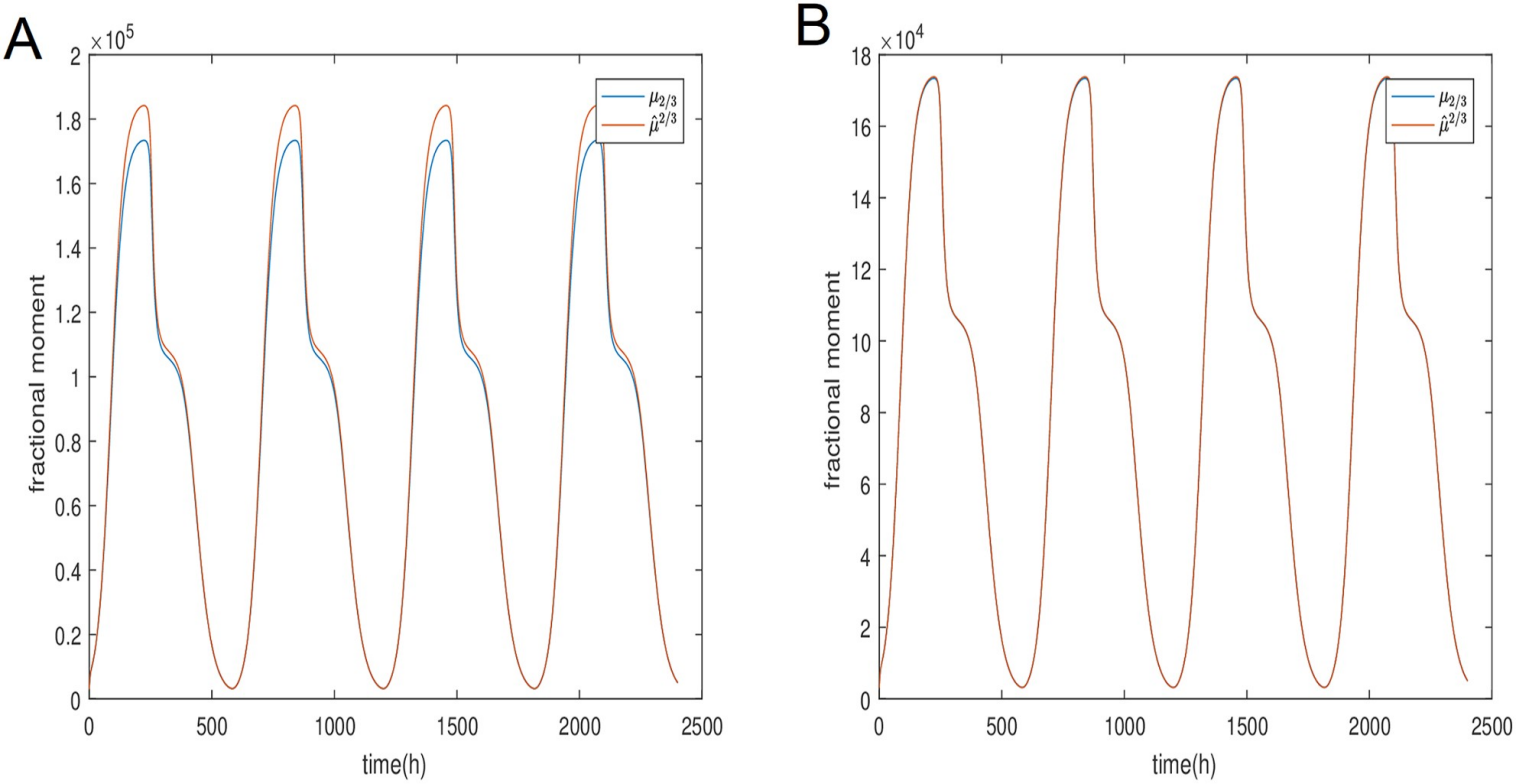
Comparison of the *μ*_2/3_ fractional moment for population y derived directly from (31) and approximated from (28). (A) Comparison of two solution for the fractional moment of the y population with *β* = 0.01. (B) Comparison of two solution for the fractional moment of the y population with *β* = 0.001.

We can see in Fig 10 that for higher values of *β* the comparison is not average but with decreasing *β* the two plot are seemingly identical. Hence, the approximation is very good.

## Discussion

In this paper we have taken a different angle of approach to the classical two populations with one substrate in a chemostat system using population balance equations instead of ODE. We wanted to investigate under which conditions can more complex models allow for a stable coexistence state that would arise solely from internal interactions. From our work it seems that one of the simplest cases where coexistence is stable is when an adaptive response to one of the populations is included. That response emerges from the ability of one of the populations to sense their biomass and adapt when it crosses some critical value by changing the mean mass of division. By formulating two models for this adaptive response competition, one semi-equation based and the other purely equation based whose dynamics are very similar, we showed that, with realistic growth parameters and dilution rate, two steady-states are possible depending on whether or not there is any delay in the sensing. The stability of these steady-states was explored through different means, namely stochastic simulations and parameters sweep. Finally, building on the work of Alexiadis et. al. [11] we were able to explain how oscillations appears in biomass equations.

Our exploration of the steady-state in the more complicated setting of PBE started with as few assumptions as possible. Our aim was to find what is the simplest way in which we can find some stable coexistence. We started with the simple simulation of the PBE model without any extra assumptions where no coexistence was found. Moving from that we carefully altered the parameters such that one population will have a higher growth rate for low mass values and the other will have a higher growth rate for higher mass values, hoping that coexistence will occur through the splitting of the distribution of the two populations, meaning that one population would gather in the lower mass spectrum and the other in the higher. That also failed so in order to “aid” the populations in achieving that we proceeded with giving them different mean masses of division. The cells that grew faster for low mass would divide earlier into smaller cells and the cells that grew faster for high mass would divide later into larger cells. Again that failed due to the fact that the substrate would eventually stabilize in a value where there would be a clear winner. Our final failed attempt, and our first move towards some sort of an adaptive response, was to introduce a division probability for population y that was a double Gaussian and hence had two peaks, one at the same mean mass of division as population x and the other at a lower mean mass. These failed attempts made it clear that it was not obvious how an adaptive response can be introduced without the cells being able to detect and take into account changes in their total populations (biomass). Hence the simplest case we came up with was the mechanism deployed in this paper.

As mentioned two steady-states were observed, one oscillatory and one non-oscillatory depending on the inclusion or expulsion of delay. In needs to be mentioned that although from a theoretical standpoint both are interesting, from a biological one the inclusion of delay is much more realistic. An instant sensing of environment would be almost impossible especially when information needs to be gathered about the total population. In addition a cell could not have been able to change its internal mechanisms immediately and there would have been an intermediate interval of reaction to the initial sensing. These two factors would contribute to the appearance of a delay in the response of a cell to its changing environment.

In addition to being more realistic, the oscillatory case raises a lot of interesting questions. One of them is how the oscillations appear in the biomass which is seemingly independent of the division rate. The plots of Figs 8 and 9 show how the dependence of the biomass on the division rate changes with changing mass dependence of the growth rates. This is formally understood through the appearance of the fractional derivative in the biomass which can be approximated by a formula that connects it to the division rate through the number of cells. The biggest significance of this observation is perhaps that it highlights the limitations of the classical model for the chemostat. If the trivial growth rate mass dependence was used these oscillations would not have been possible and as a result neither would the oscillatory coexistence of the populations. Any dependence other than the trivial is not readily translatable from PBE to ODEs.

In more complex models such as PBEs it is easy and natural to introduce various mechanisms such as the adaptive response introduced here. Since division and birth are explicitly modelled a number of different assumptions can be made and their dynamics explored. That is in contrast to simpler ODE models where further assumption would need to be made and the form of functions, that would produce a similar behaviour to PBEs, needs to be intuitively guessed. We believe that this as well as the argument in the previous paragraph make the case in favour of extending classical problems through the used of more complex mathematical tools as a lot of the phenomena observed in real life can only be captured by accounting for the underlying complexity and multi-scale interactions. Including adaptation mechanisms and changes in the division of bacterial cells has been done in a very similar setting exploring the fitness of mutant bacterial populations in a chemostat [12].

Finally, although we found no documented case of a one-celled organism with that specific population-level adaptive response mechanism it is not completely unrealistic to assume that this sort of behaviour might exist in similar form as bacterial cells are capable of changing their physiology in response to changes in their environment and are able to do so using population-level sensing and criteria. For example it was hypothesized that under competition the annual grass Bromus Madritensis might change the mean mass of its seedlings [13]: what was observed though was that the variation of the seedling mass becomes narrower instead while the mean mass stays the same. Despite the fact that this paper was dealing with multicellular organisms we can see a clear resemblance of the adaptive response, which changes characteristics of the offspring as a response to competition. Additionally, the fact that bacterial cells can change their shape due to predators, immune response and other threats [14] is well documented. The threat more related to our project would be nutritional stress which is considered a major reason of microbial cell shape changes [15]. A well documented case is that of *Actinomyces israelii* which undergoes filamentation due to the lack of essential nutrients and returns to it’s rod-like shape when the nutrients are present [16]. Moreover, quorum sensing has been well established and documented as a method of bacterial cells communicating and making population level decisions as a response to changes in their environment [6].

## Conclusion

Our work has been on the investigation of coexistence in a chemostat from a different modelling aspect and an effort to identify the path of least assumptions to achieve that. That led to the use of PBE models and the assumption of an adaptive response mechanism that only affects the mean mass of division of the cell population that deploys it. With that we were able to show that coexistence is possible and stable and that depending on the biological premises this can have different forms, an oscillatory and a non-oscillatory form. The stability is proven through stochastic simulations and parameter sweeps and we indeed observe that the steady states are reached for a wide range of noise intensities as well as a wide range of two important parameters, the dilution rate and response time.

It is important to mention some of the limitations of our work. Despite the fact that different growth parameters were tested and very similar results were found, we did not perform a exhaustive sensitivity analysis based on these parameters. Moreover, for the oscillatory case high growth rate parameters are important as otherwise the oscillation will occur in a much longer timescale which could be unrealistic. Additionally, bacteria parameters were used, in order to simulate realistic chemostat conditions, despite the fact that we would expect a more complex behaviour and an adaptive response from eukaryotic cells. Finally, although a chemostat environment might hinder such population-wide sensing mechanisms it is not a completely unrealistic setting as for lower dilution rate values it could capture the effects of a steady environment where a limited source of food is replenished at a specific rate which affects the dynamics and populations have very similar death rates.

We conclude this paper by mentioning possible extensions to this work. These can consist of performing an extended parameter sweep, including more parameters, to detect when these steady-states are lost and what are the conditions that make them appear, explore how the shape of the division rate of population y affects the dynamics and how steeper or smoother transitions change the behaviour of the system and perhaps explore more mechanisms which could induce a coexistence for the two populations in the search for a potential coexistence through even fewer or simpler assumptions. These mechanisms can include population gradient sensing where cells detect whether or not their population is decreasing instead of sensing the exact biomass. That would eliminate the need for a critical value of behaviour shift, like the one we introduced, as well as errors regarding the accuracy with which cells can “measure” the biomass which are not taken into account here. Finally, as mentioned in the previous section competition adaptation through shape change can be a viable and realistic assumption that could potentially lead to coexistence.
